# Modelling cost-effectiveness of syphilis detection strategies in prisoners: exploratory exercise in a Chilean male prison

**DOI:** 10.1186/s12962-021-00257-9

**Published:** 2021-01-23

**Authors:** Carla Castillo-Laborde, Pedro Gajardo, Manuel Nájera-De Ferrari, Isabel Matute, Macarena Hirmas-Adauy, Pablo Aguirre, Héctor Ramírez, Daniel Ramírez, Ximena Aguilera

**Affiliations:** 1 Centro de Epidemiología y Políticas de Salud, CEPS. Facultad de Medicina, Clínica Alemana, Universidad del Desarrollo, Av. Las Condes 12438, Lo Barnechea, 7710162 Santiago, Chile; 2 Departamento de Matemática, Universidad Técnica Federico Santa María, Av. España 1680, Valparaíso, Chile; 3 Instituto Oncológico, Fundación Arturo López Pérez, Rancagua 878, Providencia Santiago, Chile; 4 Departamento de Ingeniería Matemática and Centro de Modelamiento Matemático (AFB170001 - CNRS UMI 2807), Universidad de Chile, Beauchef 851, Santiago, Chile

**Keywords:** Syphilis, Modelling, Screening, Rapid test, Cost-effectiveness

## Abstract

**Background:**

Syphilis, together with other sexually transmitted infections, remains a global public health problem that is far from controlled. People deprived of liberty are a vulnerable population. Control activities in prisons rely mostly on passive case detection, despite the existence of affordable alternatives that would allow switching to active case-finding strategies. Our objective was to develop a mathematical modelling framework for cost-effectiveness evaluation, from a health system perspective, of different approaches using rapid tests for the detection of syphilis in inmates' populations and to explore the results based on a Chilean male prison population.

**Methods:**

A compartmental model was developed to characterize the transmission dynamics of syphilis inside a prison with the ongoing strategy (passive case detection, with VRDL + FTA-ABS), considering the entrance and exit of inmates over a 40 year period. The model allows simulation of the implementation of a reverse algorithm for the current situation (rapid test + VDRL), different screening strategies (entry point, massive periodically; both with rapid test + VDRL) and treatment of detected cases. The parameters for the exploratory exercise were obtained from systematic searches of indexed and grey literature and field work (EQ-5D questionnaire application and key actors interviews). Probabilistic sensitivity analysis was conducted to account for uncertainty in relevant parameters.

**Results:**

The proposed framework allows the evaluation of different detection strategies. In this study, all the strategies were cost-effective in the baseline scenario when considering an ICER threshold of 1 Chilean GDP per capita (US$15,000). The strategies most likely to be cost-effective (over 80% probability) were: current situation with reverse algorithm, entry point screening and mass screening every two years; the latter was the most effective, achieving the lowest prevalence (0.7% and 1.7% over the period versus the 3% prevalence in the current situation).

**Conclusions:**

Mathematical modelling that considers the performance of different tests and detection strategies could be a useful tool for decision making. The exploratory results show the efficiency of adopting both the use of the rapid tests and performing active case detection to significantly reduce the burden of syphilis in Chilean prisons in the near future.

## Background

Sexually transmitted infections (STIs), syphilis among them, remain a public health problem that is far from controlled. According to the World Health Organization (WHO), about 6 million new cases of syphilis occur annually worldwide [1].

In prisons, there is a higher burden of syphilis than in the general population [2, 3], with a prevalence of 2.89%, according to a systematic review [4]. Imprisonment may contribute to establishing high-risk sexual relations by destabilizing the individuals’ social and sexual networks. Moreover, the weakening of support networks and social cohesion during imprisonment increases the probability of STIs risk factors appearance (depression or anxiety, drug use, increase in the number of sexual partners), and it also increases the likelihood of acquiring or transmitting it later to the community [3, 5–7]. Thereby, the risk of syphilis for people incarcerated or with a history of incarceration is 1.52 times higher than for those who had never been in prison; and that risk increases along with the time of imprisonment [5]. Because of this, WHO includes prisoners in the key populations that should be the focus of interventions designed to reduce the burden of STIs [1]*.*

Case detection and treatment success are core elements of syphilis control that, when conducted promptly, systematically and effectively, can lead to reduced incidence, prevalence, and complications. Diagnosis of syphilis is difficult because the bacteria cannot be cultured, and serological tests are outdated. Serologic diagnostic tests require a two-step procedure; the classic approach begins with a non-treponemal screening test, followed by a more specific treponemal test. However, the increasing availability of new enzyme immunoassay treponemal tests, has led to an inverse algorithm, beginning with a treponemal test, using recombinants antigens, followed by a non-treponemal test to determine a titer [8].

Rapid tests for syphilis are effective compared to “classic” treponemal tests and have proven to be a particularly valuable and practical tool in case detection (e.g. it takes a few minutes, and can be used by untrained staff), and might contribute to improving the management of infectious diseases (i.e. diagnostic and treatment) in populations with difficult access [9–11]. Moreover, the diagnostic strategies based on rapid tests have the potential to increase access to definitive diagnosis for asymptomatic patients, preventing the development of long-term complications, and cutting the chain of disease transmission in the population [12].

The above notwithstanding, little is known about syphilis in a penitentiary context, and the most widespread practice for identifying cases is passive case detection, despite the existence of relatively less expensive alternatives that would allow for switching to active case-finding strategies. As a first approximation, and using the limited available information, we developed a mathematical model of syphilis dynamics in prisons to explore the impact of several syphilis control strategies on the prevalence of syphilis on the inmate´s population. Mathematical modelling that considers the performance of different tests and detection strategies might be a useful tool for decision making in this context. Our objective was to develop a mathematical model framework for cost-effectiveness evaluation, from a health system perspective, of different approaches using rapid tests for the detection of syphilis in inmates' populations. We then used this framework to explore the results based on a Chilean male prison population, a context with a general prevalence of 0.8% in 20 to 49 years old males [13], reaching 3% in male prisons [14].

## Methods

### Mathematical modelling

Modelling the cost-effectiveness evaluation of different strategies for the detection of syphilis in inmates' populations requires the following three components:

i. A mathematical model representing the evolution of the disease in prison population: For our case study (a Chilean inmate population) and based on the data available, we consider the following three assumptions: (1) Currently the disease is in a steady state in both the general population (outside the prison) and in the inmate population; (2) The population is isolated, that is, inmmates do not have sexual contacts with people from outside the prison or with workers of the prison and; (3) The number of innmates is constant. Considering these assumptions, we adapt the model published by Garnett et al. to a prison context [15]. The result is a compartmental model, a system of Ordinary Differential Equations (ODE), with the inmates distributed into six groups: Susceptible (S), Infected in stage 1 $({Y}_{1})$, Infected in stage 2 (${Y}_{2}$), Latent infection (L), Tertiary syphillis (T) and Immune (I). See Additional file 1: Appendix S1 for model details.
ii. Representation of different strategies to be evaluated: In the mathematical model previously described, we included the action of different detection strategies to be evaluated (including treatment of confirmed cases). These strategies affect the evolution of the disease, modifying transitions of inmates between different disease stages (compartments). The different strategies can be divided into two broad categories: (1) passive detection, that examines syphilis suspect cases among persons who spontaneously seek health care, and (2) active case finding, through the screening of apparently healthy people. The later could be performed at the entrance to imprisonment (entry screening), or once inside the prison, through periodic mass screening. The modelling of how detection strategies affect the disease evolution must consider the sensitivity and specificity of each involved test. The tests included in the analysis are Rapid Test (RT), Non Treponemal Test (VDLR), and Treponemal Test (FTA-ABS). Depending on the combinations of the screening strategy, the detection tests, and the order in which they are applied, we simulated and compared the following strategies:

- Strategy 0 (current situation, passive detection): Classic Non Treponemal Test upon spontaneous consultation (inside the prison), based mainly on symptoms, followed by a Treponemal confirmation Test;
- Strategy 1 (entry screening): Systematic detection of a large proportion of prisoners entering the prison using the Rapid Test, followed by a non-treponemal test to determine a titer;
- Strategy 2a, 2b, 2c and 2d (mass screening): Periodic mass screening of inmates using the Rapid Test, with three different frequencies (i.e., every 1, 2, 5, or 10 years), in each case followed by a non-treponemal test to determine a titer;
- Strategy 3 (current situation, reverse algorithm): Detection test upon spontaneous consultation (inside the prison) using the Rapid Test, based mainly on symptoms, followed by a non-treponemal test to determine a titer.
  Strategies 1 and 2 are incremental to Strategy 0, considering the potential detection of spontaneous visitors to health centers.
  In Additional file 1: Appendix S1 we include the modelling of these strategies representing the downstream effects of each of them in the disease evolution. For instance, when new inmates who test positive for syphilis enter the prison they have to be assigned to different stages of the disease, which would be different if considering entry screening or the other strategies.

iii. Model of the cost and health outcomes associated with each strategy: With the previous two components, the model estimates the number of infected and non infected inmates as the direct result of the detection and treatment according to each of the strategies. Therefore, the effectiveness of the strategies, that we assessed using quality adjusted life years (QALY), are derived from these outputs, while the costs are derived from the number of tests applied (detection and confirmation) and the number of treatments delivered. The mathematical modelling of costs and health outcomes for our case study are described in Additional file 1: Appendix S1.

### Parameters, background, and source of data

In order to run the model previously described and to proceed with the cost-effectiveness evaluation of strategies considered, a next step is to identify different parameters involved in the model. Based on the available data, in this procedure several assumptions need to be considered.

In our case study, most of the parameters, mainly those related to the disease dynamic and test performance were obtained from systematic searches of indexed (PubMed, Cochrane, Scielo) and non-indexed (Chilean Ministry of Health reports, clinical guidelines) literature, complemented with epidemiological textbooks. Based on previous work that measured the syphilis prevalence in two Chilean prisons using rapid tests for detection and non-treponemal tests for confirmation [14], we calibrated the model taking into account the available values for parameters in order to obtain a stationary 3% prevalence as an outcome for the Strategy 0 (see Table A2.1 in Additional file 1: Appendix S2). For the values of some key parameters (transmission probabilities, number of sexual partners, and inmates turnover) we proceeded as follows: (i) we assumed that the current situation in the prison, represented by the initial condition used in the dynamics, is at equilibrium; (ii) for some choice of the mentioned parameters, we computed the corresponding steady state of the system describing the disease dynamics and we compared it with the initial condition (square norm of the difference); (iii) we chose the set of parameters in ranges indicated by the references in Table A2.1 (Additional file 1: Appendix S2), whose corresponding steady state best approximates the initial condition (with respect to the square norm criterium) –i.e. a least-square parameter estimation, considering obtained steady states. This procedure is explained in Additional file 1: Appendix S1.

Test performance and treatment effectiveness were also obtained from the literature review. Table A2.2 in Additional file 1: Appendix S2 shows the sensitivity and specificity values for the detection and confirmation tests. Treatment depended on the disease stage, using penicillin in different doses and routes of administration, and tetracycline for the allergic (2%). We also assumed a re-treatment probability of 10% [16, 17].

The health outcomes associated with uninfected and infected inmates were assessed using QALY. In the case of infected individuals, the QALY were measured through the application of the EuroQol-5 Dimension questionnaire (EQ-5D), in its 3L version provided in Spanish by Euroqol Research Foundation (after registration to use and authorization). We used a convenient sample of 29 infected inmates from the prison of Arica city, interviewed in January 2016 as part of the prevalence study mentioned before [14], and additionally 67 infected patients from a hospital outpatient clinic of sexually transmitted infection (UNACCES) of the “Sótero del Río” Hospital, in Santiago, interviewed between March and November 2017. The EQ-5D profiles were valued based on a previous study commissioned by the Superintendencia de Salud [18] in order to obtain the quality of life coefficients by stage. The value for infected individuals was calculated as the mean value for the combined sample for the 96 individuals (with no significant differences between the two sub-samples mean values), resulting in a value of 0.737 (see Table A2.2 in Additional file 1: Appendix S2). In the case of uninfected inmates, “perfect health” was assumed, with a value of 1 according to the EQ-5D profile valued by the same study [18].

**Fig. 1:**
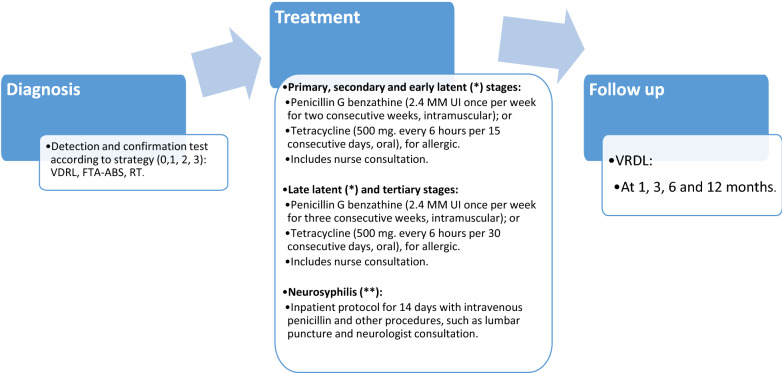
**Use of resources.** (*) Early latent represents 45% of the latent cases and late latent 55% of these cases. (**) The analysis also considers a chance of developing neurosyphilis at every stage of the disease (0.9% primary, 3.8% secondary, 3.1 early latent and 7.1% in late stages) [23]

For both detection and treatment costs, Chilean protocols and clinical guidelines were reviewed [19–22] and unstructured interviews were carried out with key informants (Ministry of Health-MoH–STI Responsible; Institute of Public Health–STI Laboratory Responsible; Gendarmeria Chile–Head of Health Department; UNACESS Sótero del Río–health professionals STI Unit; Arica Prison –health officer), in order to identify the usual practice regarding the detection algorithm, type of test utilized for detection and confirmation, and treatment for each stage of the disease (see Fig. 1). In addition, in order to assign monetary values to the resources that were identified and measured (see Table A2.2 in Additional file 1: Appendix S2) we used Chilean National Health Fund (FONASA) fees for health services and centralized national procurement agency (CENABAST) prices for medicines.

For the cost-effectiveness analysis we estimated the incremental cost-effectiveness ratio (ICER), comparing the discounted cost and health outcomes obtained from the model for each of the strategies (1, 2 and 3) with strategy 0 as a reference. The coverage for both the entry point strategy and mass screening was 80%, the time period was 40 years, and a discount rate of 3% was applied according to the Chilean guidelines for cost-effectiveness analysis [24].

Finally, to take into account the uncertainty associated with relevant parameters (see Table A2.2 in Additional file 1: Appendix S2), a probabilistic analysis (1000 iterations) was performed. This analysis used ranges for the values according to the literature review, and statistical distribution based on a published cost-effectiveness analysis [25].

## Results

The proposed mathematical modelling framework, which considers the performance of the tests to be applied, allows the evaluation of different detection strategies. As a result, the model estimates the number of infected and not infected inmates for a given strategy.

**Fig. 2:**
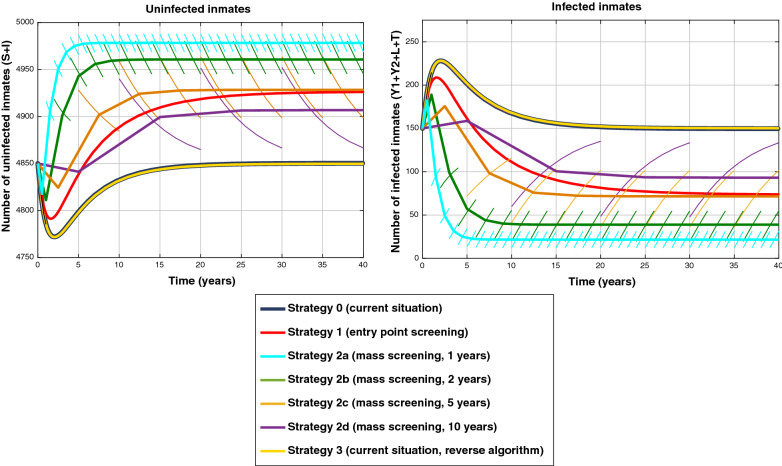
Infected and not infected individuals over a 40 year period, according to each simulated strategy

For the exploratory exercise, over the 40 year period and 5,000 inmates, these numbers are presented in Fig. 2. As expected from the calibration, “strategy 0”, passive detection, shows a stationary number of infected close to 150 (3% prevalence), while “strategy 3” (passive detection with reverse algorithm) shows similar results. All the active case detection strategies (1 and 2) result in lowering syphilis prevalence of varying degrees. Mass screening every year (“strategy 2a”) is the most effective, achieving the lowest prevalence over the period (under 1% or less than 50 infected per year, varying on average between 0.4% and 0.8% over the period), while “strategy 2d” (every ten years), led to a prevalence varying on average between 1.6% and 2.9%. On the other hand, the entry screening (“strategy 1”) led to a 1.6% prevalence over the 40 years.

**Fig. 3:**
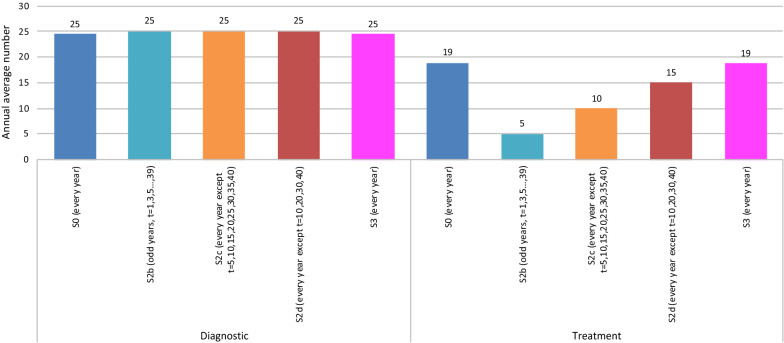
Annual average number of diagnostic tests and treatments, Strategy 0, Strategy 3 and Strategy 2 (no mass screening years)

**Fig. 4:**
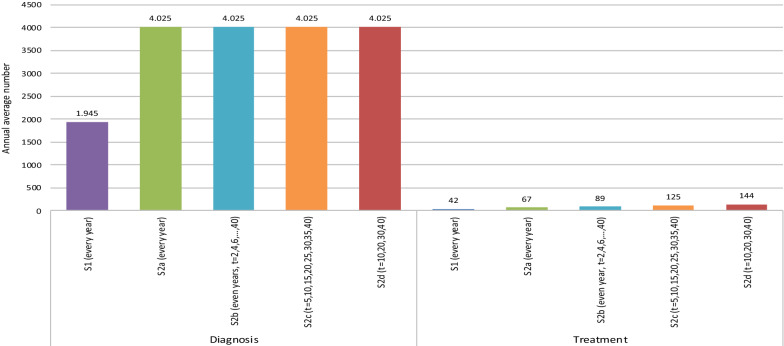
Annual average number of diagnostic tests and treatments, Strategy 1 and Strategy 2 (mass screening years)

The number of diagnosis and confirmation tests performed, as well as treatments performed every year under each of the strategies are shown in Figs. 3, 4. For strategy 0 and strategy 3 the number of tests and treatments were similar, while strategy 1 had a uniform yearly pattern. For strategy 2 the testing and treatment pattern depended on the periodicity of the mass screening (similar to strategies 0 and 3 during no mass screening years and much higher during mass screening years).

Table 1 presents, for each strategy, the cost and effectiveness, as well as the ICER compared to the current situation (“strategy 0”). When considering an ICER threshold of 1 Chilean GDP per capita (near to US$ 15,000), as recommended by the Chilean cost effectiveness guidelines [24], the analysis of the baseline scenario suggests that all the strategies are cost-effective. The most effective strategy over the study period is the “strategy 2a” (mass screening every year), with an incremental 843.3 QALYs, being also the most expensive (incremental cost of US$ 2,832,838). The most cost-effective strategy is mass screening every 10 years.

**Table 1:** ICER results (baseline scenario)

	Strategy 0 (current situation)	Strategy 1 (entry point screening)	Strategy 2a (mass screening, 1 year)	Strategy 2b (mass screening, 2 years)	Strategy 2c (mass screening, 5 years)	Strategy 2d (mass screening, 10 years)	Strategy 3 (current situation, reverse algorithm)
Total QALYs	116,250.7	116,583.3	117,094.0	116,930.0	116,641.8	116,443.6	116,247.8
Total costs (^a^)	$56,050	$261,996	$2,832,838	$888,054	$243,926	$120,481	$46,281
Incremental QALYs		332.6	843.3	679.3	391.1	192.9	– 2.9
Incremental costs (^a^)		$205,946	$2,776,788	$832,004	$187,876	$64,431	$-9,768
ICER (^a^)		$619.20	$3,292.61	$1,224.8	$480.40	$334.00	$3,382.4

^a^US$ 2017

**Fig. 5:**
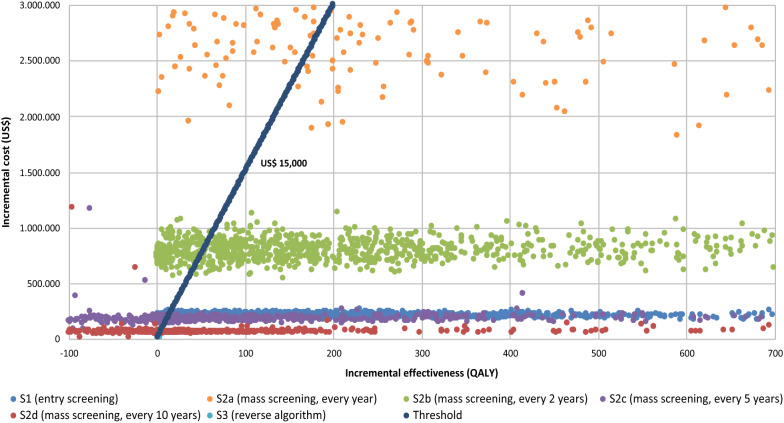
Probabilistic sensitivity analysis results

Finally, Fig. 5 shows the results of the probabilistic sensitivity analysis for the 1,000 performed iterations, according to the value ranges and probabilistic distributions presented in Table A2.2 (Additional file 1: Appendix S2). The probabilities for the ICER to be below the 1 GDP per capita threshold were: 94.3% for “strategy 1”; 66.7% for “strategy 2a” (every year); 83.0% for “strategy 2b” (every two years); 48.1% for “strategy 2c” (every five years); 41.11% for “strategy 2d” (every ten years); and 95.3% for “strategy 3”. This suggests, given the assumptions, that the strategies associated with a higher likelihood of being cost-effective would be the reverse detection algorithm (“strategy 3”), the entry point screening (“strategy 1”) and the mass screening every two years (“strategy 2b”).

## Discussion

The WHO Global Strategy on Sexually Transmitted Infections 2016–2021 recognizes syphilis as one of the diseases that needs to be addressed immediately. Prisoners, as part of the high risk and vulnerable populations should also be targeted, with early diagnosis followed by treatment as a recommended intervention [1]. Studies on diagnosis and treatment strategies in penitentiary settings are limited. However, it is recognized that active case-finding, when compared to passive case-finding, promotes early diagnosis and allows the treatment of affected individuals, even in asymptomatic stages, therefore, preventing disease transmission both within the prison and to the general population [26–28]. The studies have also pointed out that the use of less invasive and rapid tests has proven to be successful in prison environments, resulting in increased application of testing [26, 29].

This study shows the feasibility of different interventions and their effects on syphilis dynamics in a prison environment. These methods can be adapted to more realistic models where knowledge and data are available, in other contexts (e.g. female prisons), or for use in evaluating the cost-effectiveness of a combination of strategies (e.g. a combination of entry point screening and periodic massive screening).

In line with the guidelines, and as a complement to the available evidence, our exploratory exercise shows the convenience of adopting both the use of the rapid tests and performing active case detection to reduce the burden of syphilis in Chilean prisons. These strategies lead to a reduced prevalence at a cost that falls below the Chilean ICER threshold of 1 Chilean GDP per capita (near US$ 15,000).

According to our findings, passive case detection using a rapid test with the reversal algorithm reached the highest certainty in cost-effective analysis; however, it is not effective in reducing syphilis prevalence in the long term. Entry screening and mass screening every two years also have high certainty in cost-effective analysis, and both led to an effective reduction in syphilis prevalence. Passive case detection strategies, either the current situation or the reversal algorithm, were found to maintain the prevalence of syphilis around 3% over the period of 40 years, while the active case detection strategies analyzed in this study reduce the prevalence to levels close to 1%.

While the current syphilis control activities in Chilean prisons, based on passive case detection, have a high prevalence, our study identifies alternatives, of straightforward application (even in contexts of difficult access) and with an affordable and short course detection/treatment, that can help to control the situation. Therefore, active strategies may be warranted.

One of the main limitations of the proposed approach is the strong dependence on the availability of local information. In the case of syphilis in Chile, and specifically in Chilean prisons, epidemiological sources are scarce (for instance, time series are not available), which result in the triangulation of the available information based on assumptions relying on expert judgement.

The above notwithstanding, the construction of mathematical models that consider the performance of different tests and detection strategies can be a useful tool for decision making.

## Conclusions

Two main conclusions can be drawn from this study. First, from the mathematical perspective, modelling demonstrates that the performance of different tests and detection strategies might be a useful tool for decision making, with a broad spectrum of areas in which these types of approaches can be used. Second, from a decision making standpoint, the exploratory results show the efficiency of adopting both the use of the rapid tests and performing active case detection to significantly reduce the burden of syphilis in Chilean prisons.

## Supplementary Information

**Additional file 1: Appendix S1.** Mathematical models of the syphilis dynamics, costs, and health outcomes under different intervention strategies. **Appendix S2.** Tables of parameters: Exploratory exercise in a Chilean male prison.

## Data Availability

The Mathematical models of the syphilis dynamics, costs, and health outcomes under different intervention strategies and the value for the parameters used in the exploratory exercise are available in Additional file 1: Appendix S1, 2 respectively (electronic supplementary material).
