# Supplementary material for: Modelling cost-effectiveness of syphilis detection strategies in prisoners: exploratory exercise in a Chilean male prison
Source: Cost Eff Resour Alloc. 2021 Jan 23;19:5. doi: 10.1186/s12962-021-00257-9 (PMC7825166; doi:10.1186/s12962-021-00257-9)
Supplement: Supplementary file 1 — Additional file 1: Appendix S1. Mathematical models of the syphilis dynamics, costs, and health outcomes under different intervention strategies. Appendix S2. Tables of parameters: Exploratory exercise in a Chilean male prison. [file 12962_2021_257_MOESM1_ESM.pdf]

Cost Effectiveness and Resource Allocation
(Additional file 1)

Modelling cost-effectiveness of syphilis detection strategies in prisoners: exploratory exercise in a Chilean male prison

Electronic supplementary material
Additional file 1

C. Castillo-Laborde · P. Gajardo · M. Nájera-De Ferrari · I. Matute · M. Hirmas · P. Aguirre · H. Ramírez · D. Ramírez · X. Aguilera

Received: 5 August 2020 / Accepted: 2 January 2021

Appendix S1. Mathematical models of the syphilis dynamics, costs, and health outcomes under different intervention strategies

The results exhibited in the paper, for the exploratory exercise in a Chilean male prison, were obtained with detailed mathematical models of the disease dynamics, costs, and health outcomes under the intervention strategies, as mentioned in the Section *Materials and methods* (*Mathematical modelling*). The purpose of this appendix is to describe those mathematical models and formulations, that will use the parameters presented in tables A2.1 and A2.2 in Appendix S2. This appendix also describes the calibration procedure of some key parameters. All the mathematical expressions, fully described in this document, were numerically obtained with routines implemented in MATLAB. In particular, ordinary differential equations (ODE with initial values) were solved with the *ode45* Runge-Kutta method.

C. Castillo-Laborde (corresponding author E-mail: carlacastillo@udd.cl), M. Nájera-De Ferrari, I. Matute, M. Hirmas, X. Aguilera
Facultad de Medicina, Clínica Alemana, Universidad del Desarrollo, Santiago, Chile

P. Gajardo, P. Aguirre, D. Ramírez
Departamento de Matemática, Universidad Técnica Federico Santa María, Valparaíso, Chile

H. Ramírez
Departamento de Ingeniería Civil Matemática and Centro de Modelamiento Matemático (CNRS UMI 2807), Universidad de Chile, Santiago, Chile

This appendix is organized in four sections: Model of the disease dynamics under different intervention strategies; Costs of intervention strategies; Health outcomes of intervention strategies; and Calibration of key parameters.

1 Model of the disease dynamics under different intervention strategies

As presented in the Section *Materials and methods*, strategies to be considered are related to the application of different screening methods, where the treated subjects are all the detected cases (true and false positives). Screening-treatment procedures can be applied to inmates entering the prison (at the entry point) or to a fraction of its population inside the prison. The screening tests to be considered are: Rapid Test (RT), Non Treponemal Test (VDRL), and Treponemal Test (FTA-Abs). Depending on the moment when the screening tests are applied (at entry point or inside the prison) and on the order in which they are applied, the strategies considered are the following.

– **Strategy 0 (current situation, passive detection)**: Screening of inmates inside the prison with a Non Treponemal Test, based mainly on symptoms. This is followed by a confirmation Treponemal Test applied to detected cases;
– **Strategy 1 (entry screening)**: Screening of a large percentage of inmates entering the prison using the Rapid Test;
– **Strategy 2a, 2b, 2c, and 2d (mass screening inside the prison)**: Mass screening of inmates inside the prison using the Rapid Test, with three different frequencies: every 1, 2, 5, and 10 years, respectively;
– **Strategy 3 (current situation, reverse algorithm)**: Screening of inmates inside the prison using the Rapid Test based mainly on symptoms.

In strategies 1, 2, and 3, after the first test, a Non Treponemal Test is applied to confirm detected cases. In strategies 1 and 2 the procedure described in Strategy 0 continues being applied simultaneously. In every strategy, all confirmed cases are treated. In the model description, we will use the notation $\mathcal{E} \in \{0, 1, 2, 3\}$ for referring to a strategy in particular.

Concerning the model of the disease dynamics under the described intervention strategies, we adapt the model proposed in [4] to a prison context, considering the data available for this study. Thus, the model proposed consists of a compartmental model, where the inmates are distributed into 6 groups corresponding to different stages of the disease: Susceptible (S), primary (Y_1), secondary (Y_2), latent (L), tertiary (T), and immune (I). As usual in controlled dynamical systems, these groups of stages are called state variables. The evolution of these state variables

is described by the following system of ordinary differential equations:

$$\begin{cases} \dot{S} &= \Lambda_S(\xi_\mathcal{E}) + \tau_{Y_1}(\mathcal{E})Y_1 + \phi(\mathcal{E})\tau_{Y_2}(\mathcal{E})Y_2 + \sigma_I I - \left(c_1\alpha\frac{\beta_1 Y_1}{N} + c_2\frac{\beta_2 Y_2}{N}\right)S - \mu S \\ \dot{Y_1} &= \Lambda_{Y_1}(\xi_\mathcal{E}) + \left(c_1\alpha\frac{\beta_1 Y_1}{N} + c_2\frac{\beta_2 Y_2}{N}\right)S - (\sigma_{Y_1} + \tau_{Y_1}(\mathcal{E}) + \mu)Y_1 \\ \dot{Y_2} &= \Lambda_{Y_2}(\xi_\mathcal{E}) + \sigma_{Y_1}Y_1 - (\sigma_{Y_2} + \tau_{Y_2}(\mathcal{E}) + \mu)Y_2 + \rho_\mathcal{E}(Y_2(t))L \\ \dot{L} &= \Lambda_L(\xi_\mathcal{E}) + \sigma_{Y_2}Y_2 - (\rho_\mathcal{E}(Y_2(t)) + \sigma_L + \tau_L(\mathcal{E}) + \mu)L \\ \dot{T} &= \Lambda_T(\xi_\mathcal{E}) + \sigma_L L - (\tau_T(\mathcal{E}) + \mu)T \\ \dot{I} &= \Lambda_I(\xi_\mathcal{E}) + (1-\phi(\mathcal{E}))\tau_{Y_2}(\mathcal{E})Y_2 + \tau_L(\mathcal{E})L + \tau_T(\mathcal{E})T - (\sigma_I + \mu)I. \end{cases} \tag{A1.1}$$

The above system is represented also in Figure A1.1 below. Since model (A1.1) is an adaptation of that introduced in, [4] in this section we only describe the new components and features. Notice that system (A1.1) depends explicitly on the chosen strategy $\mathcal{E} \in \{0, 1, 2, 3\}$. From the mathematical viewpoint, Strategies 2a, 2b, 2c, and 2d are basically the same, so, we analyze them together. For a given strategy $\mathcal{E}$, the value $\xi_\mathcal{E} \in [0, 1]$ represents the fraction of new inmates entering the prison to whom the Rapid Test is applied at a constant rate.

Model (A1.1) allows the simulations of infection, detection and treatment of cases for different strategies introduced in the Section *Materials and methods*. Susceptible individuals (S) can be infected by diseased individuals in primary (Y_1) or secondary (Y_2) stages (see Figure A1.1, transition 1). The transitions from different disease stages, following the natural history of Syphilis, are: from primary to secondary (transition 2), from secondary to latent (L) (transition 3), from latent to tertiary (T) (transition 4), and from immune (I) to susceptible (transition 5). Notation-wise, the transition from a disease state, say X, to the following stage is occurring at rate σ_X, where σ_X^{-1} represents the mean duration of stage X. In addition, a transition from latent state L to secondary state Y_2 is considered (transition 6), due to the relapse of latent cases to an infectious syphilitic state.

Treatments inside the prison, for individuals at stage X of the disease, are represented by the treatment rate τ_X. These rates depend on the strategy $\mathcal{E}$ considered. Treated inmates at the primary stage of the disease reenter into the susceptible class (transition 7). Treated individuals at the latent or tertiary stages of the disease acquire immunity (transitions 10 and 11). Treated inmates at the secondary stage can reenter into the susceptible class (transition 8) or may acquire immunity (transition 9).

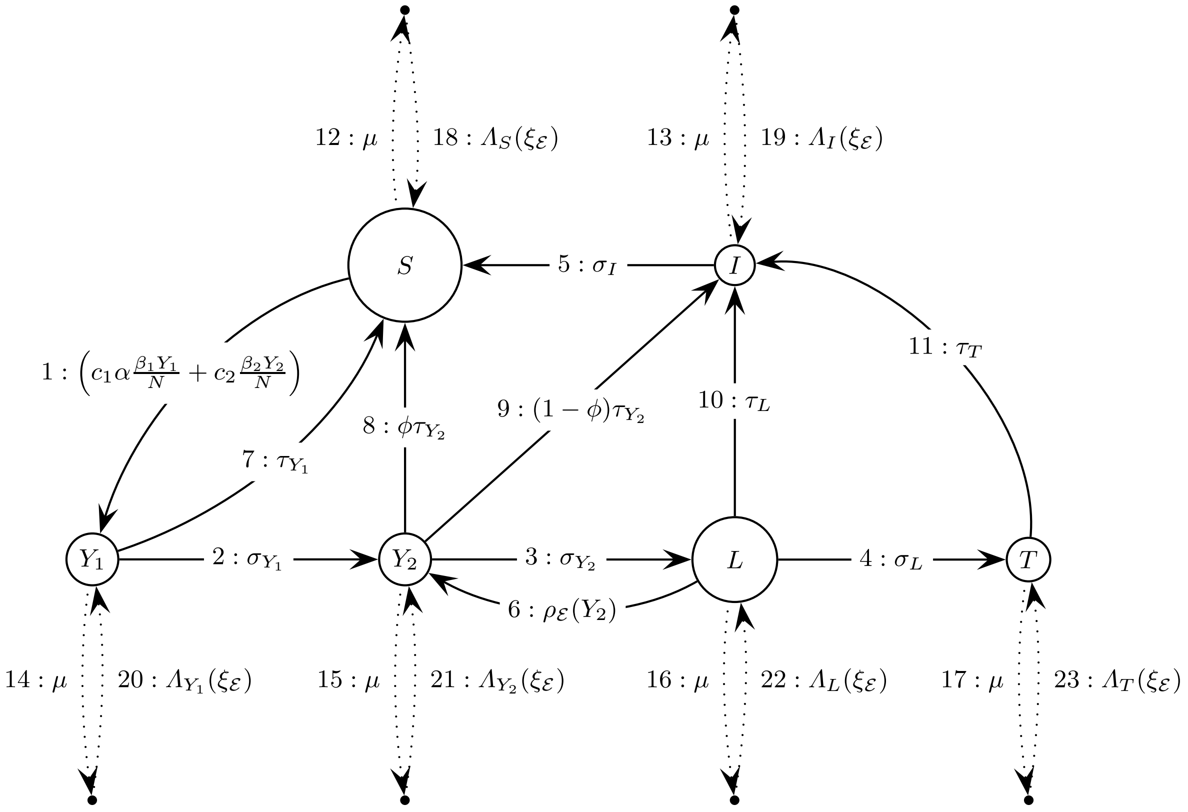

Fig. A1.1 Structure of the mathematical model for the dynamics of syphilis in prison. Each circle represents a compartment. Susceptible individuals (S), and different disease states: primary (Y_1), secondary (Y_2), latent (L), tertiary (T), and immune (I). Treatment rates from compartment X is represented by τ_X. Discharge of the prison is represented by μ and entry to compartment X (depending on the strategy $\mathcal{E}$ considered, specifically on $\xi_\mathcal{E}$) is denoted by $\Lambda_X(\xi_\mathcal{E})$.

Finally, we are assuming that the number of inmates in the prison is constant and equal to N. Therefore, entry and exit rates in and out of the prison are equal (denoted by μ). In Figure A1.1, at each compartment there is an exit flow (out of the prison) at rate μ (see flows 12-17). Nevertheless, the entries to the prison have to be distributed in different compartments, and this distribution depends on the

strategy $\mathcal{E}$ considered because it may involve the application of the Rapid Test and treatment before entering the prison. Thus, the entry flow of new inmates to a stage X is denoted by $\Lambda_X(\xi_{\mathcal{E}})$ (see flows 18-23). Moreover, the sum of all the entry rates $\Lambda_X(\xi_{\mathcal{E}})$ over all the stages X is equal to μN.

The dynamics under strategies 0, 1 and 3 ($\mathcal{E} \in \{0, 1, 3\}$) are represented by system (A1.1) with the corresponding choice of $\xi_{\mathcal{E}}$. Namely, if the Rapid Test is applied constantly to a fraction $\xi_{\mathcal{E}} \in [0, 1]$ of new inmates, strategies 0 and 3 correspond to $\xi_{\mathcal{E}} = 0$. On the other hand, Strategy 1 is represented by having $\xi_{\mathcal{E}}$ positive.

The main difference of Strategies 2a, 2b, 2c, and 2d concerns the application of the mass screening to a portion of the prison population, which is done in a very short period of time. Hence, it implies abrupt jumps in the values of state variables in (A1.1). For this reason, in Section 1.4 we describe separately these strategies ($\mathcal{E} = 2$).

From the mathematical modeling viewpoint, we recall that the objective of this study is to implement a decision model for evaluating the cost-effectiveness of different interventions. The proposed model representing the disease dynamics does not consider all the disease stages as such published in [4] and it is valid under important assumptions. This is explained by the available data to which we had access. In this regard, we are not considering interactions between inmates and the outside population, the high variability of the number of sexual partners of inmates (represented by parameters c_j), and the high variability of inmates' conviction times, represented by $1/\mu$. While some degree of realism may be spared —as in any mathematical model—, these assumptions allow us to construct a fair and tractable mathematical representation of our object of study that successfully accounts for its complexity. Namely, the contribution of the proposed mathematical model is its ability to represent the different interventions and their effects in the disease dynamics (as explained in the following sections), taking into account the performance of the applied tests, which can be adapted to more realistic models when knowledge and data are available.

Comparing model (A1.1) with the proposed in [4], we do not consider three state variables: incubation, relapse, and remission stages. To omit the incubation stage implies to consider that a proportion of inmates in the primary stage are not infectious. For this reason we have introduced the parameter α in the first and second equation in (A1.1). If $\tilde{\sigma}_E^{-1}$ and $\tilde{\sigma}_{Y_1}^{-1}$ represent the mean duration of incubation and primary stages in the model proposed in [4], we compute the proportion

of infectious individuals in primary stage in our model as

$$\alpha = \frac{\tilde{\sigma}_{Y_1}^{-1}}{\tilde{\sigma}_{Y_1}^{-1} + \tilde{\sigma}_E^{-1}} = \frac{\tilde{\sigma}_E}{\tilde{\sigma}_{Y_1} + \tilde{\sigma}_E}.$$

Considering the values of $\tilde{\sigma}_E^{-1}$ and $\tilde{\sigma}_{Y_1}^{-1}$ in [4], we obtain $\alpha = 0.622$ as specified in Table A2.1 in Appendix S2. On the other hand, the value of σ_{Y_1} was also computed taking into account the values $\tilde{\sigma}_E$ and $\tilde{\sigma}_{Y_1}$ in [4], because in our model ${\sigma_{Y_1}}^{-1}$ would represent the time in the primary stage plus an incubation time. Thus, we have ${\sigma_{Y_1}}^{-1} = \tilde{\sigma}_E^{-1} + \tilde{\sigma}_{Y_1}^{-1}$, and therefore

$$\sigma_{Y_1} = \frac{\tilde{\sigma}_{Y_1}\tilde{\sigma}_E}{\tilde{\sigma}_{Y_1} + \tilde{\sigma}_E}.$$

According to the values of $\tilde{\sigma}_E^{-1}$ and $\tilde{\sigma}_{Y_1}^{-1}$ in [4], we obtain $\sigma_{Y_1} = 4.932$ $[\text{year}]^{-1}$ as it is presented in Table A2.2 in Appendix S2.

The distribution between susceptible and immune inmates treated at secondary stage ($\phi(\mathcal{E}) \in [0, 1]$), explained in Section 1.2 below, is another parameter in (A1.1) not considered originally in [4]. This parameter is introduced because we are not considering relapse and remission stages. Although treatment rates are considered in [4], how these rates vary according each strategy is described in Section 1.1. Finally, since in [4] all new individuals are entering to the susceptible stage and we are adapting this model to a prison context, we have to consider flows of new inmates entering eventually to all stages of the disease. These flows, represented by $\Lambda_X(\xi_{\mathcal{E}})$ in (A1.1) and in Figure A1.1, depend on the applied strategy. This modeling approach is described in Section 1.3.

1.1 Treatment rates inside the prison

Treatment rates inside the prison, denoted by τ_X, correspond to flow rates from a stage X of the disease to susceptible or immune stages, due to treatments of inmates presenting symptoms. These rates depend on the distribution of inmates in each stage of the disease, which in turn depend on the applied strategy $\mathcal{E} \in \{0, 1, 2, 3\}$.

Regarding Figure A1.1 or system (A1.1), the treatment rates inside the prison are $\tau_{Y_1}(\mathcal{E})$, $\tau_{Y_2}(\mathcal{E})$, $\tau_L(\mathcal{E})$, and $\tau_T(\mathcal{E})$, where the subscript denotes the stage or variable state where treated inmates are.

Given a strategy $\mathcal{E} \in \{0, 1, 2, 3\}$, the screening process inside the prison consists in a test $d_1 \in \{\text{VDRL}, \text{RT}\}$ followed by a confirmation test $d_2 \in \{\text{VDRL}, \text{FTA-Abs}\}$.

Then, in order to compute the rates $\tau_j(\mathcal{E})$ (with $j \in \{Y_1, Y_2, L, T\}$), we need to consider the following equations:

$$\tau_j(\mathcal{E}) = s_j(d_1)s_j(d_2)\gamma_j \tag{A1.2}$$

$$\tilde{p}_j(\mathcal{E})P_j(\mathcal{E}) = s_j(d_1)s_j(d_2)\gamma_j P_j(\mathcal{E}) + (1 - e_j(d_1))(1 - e_j(d_2))(\eta - \gamma_j P_j(\mathcal{E})), \tag{A1.3}$$

where

- $s_j(d)$ and $e_j(d)$ are the sensitivity and specificity of the test denoted by $d \in \{\mathrm{VDRL}, \mathrm{RT}, \mathrm{FTA\text{-}Abs}\}$ (values indicated in Table A2.2 in Appendix S2) respectively.
- γ_j is the fraction of people in the stage j to whom the screening process is applied. We suppose γ_j does not depend on the strategy since it is mainly related to symptoms of stage j. In principle, these values are unknown, but we describe the assumptions that allow their computation.
- $\tilde{p}_j(\mathcal{E})$ is the number of treated individuals after being detected at stage j (including false positive cases), divided by the total number of people at this stage. These values are assumed to be known for strategy $\mathcal{E} = 0$ (representing the current situation at steady state) and are shown in Table A2.2 (Appendix S2).
- $P_j(\mathcal{E})$ is the prevalence of the disease at stage j inside the prison, which depends on the chosen strategy $\mathcal{E}$. These values are assumed to be known for strategy $\mathcal{E} = 0$ (representing the current situation at steady state) and are shown in Table A2.2 (Appendix S2).
- The number $\eta \in [0, 1]$ represents the proportion of inmates undergoing diagnosis inside the prison. We assume this number does not depend on the strategy considered. The value of this parameter is $\eta = 0.0049$, as indicated in Table A2.2 in Appendix S2. Nevertheless, we will allow parameter η (and others) to vary in the sensitivity analysis described in the Section *Results* (see also Figures 3 in the paper).

Since $\tilde{p}_j(\mathcal{E})$ and $P_j(\mathcal{E})$ are known for the strategy $\mathcal{E} = 0$ (at steady state), consisting in $d_1 = \mathrm{VDRL}$ and $d_2 = \mathrm{FTA\text{-}Abs}$ then, the values of γ_j (independent on the strategy), according to equation (A1.3), are given by

$$\gamma_j = \frac{1}{P_j(\mathcal{E})}\left(\frac{\tilde{p}_j(\mathcal{E})P_j(\mathcal{E}) - \eta(1 - e_j(d_1))(1 - e_j(d_2))}{s_j(d_1)s_j(d_2) - (1 - e_j(d_1))(1 - e_j(d_2))}\right) \qquad j \in \{Y_1, Y_2, L, T\}. \tag{A1.4}$$

With these values of γ_j one can compute $\tau_j(\mathcal{E})$ using equation (A1.2).

From equation (A1.4), sensitivity and specificity of tests denoted by $d \in \{\text{VDRL}, \text{RT}, \text{FTA-Abs}\}$ (values indicated in Table A2.2 in Appendix S2), and values of $\tilde{p}_j(\mathcal{E})$ and $P_j(\mathcal{E})$ indicated in Table A2.2 (Appendix S2), we obtain the values for γ_j shown in Table A1.1 below, which can then be used to compute treatment rates for strategies 0, 1, 2 and 3 using equation (A1.2), thus obtaining Table A1.2.

Notation	Value	Unit
γ_{Y_1}	0.1071	$(\text{year})^{-1}$
γ_{Y_2}	0.1145	$(\text{year})^{-1}$
γ_L	0.1918	$(\text{year})^{-1}$
γ_T	0.3658	$(\text{year})^{-1}$

Table A1.1 Values of γ_j representing the fraction of people in the stage j to whom the screening process is applied.

Notation	$\mathcal{E}=0$	$\mathcal{E}=1$	$\mathcal{E}=2$	$\mathcal{E}=3$	Unit
$\tau_{Y_1}(\mathcal{E})$	0.0652	0.0664	0.0652	0.0664	$(\text{year})^{-1}$
$\tau_{Y_2}(\mathcal{E})$	0.1115	0.1138	0.1115	0.1138	$(\text{year})^{-1}$
$\tau_L(\mathcal{E})$	0.1711	0.1717	0.1711	0.1717	$(\text{year})^{-1}$
$\tau_T(\mathcal{E})$	0.2592	0.3394	0.2592	0.3394	$(\text{year})^{-1}$

Table A1.2 Treatment rates inside the prison, after screening and confirmation tests, applied mainly to inmates presenting symptoms, for different strategies and stages of the disease.

1.2 Distribution between susceptible and immune inmates treated at secondary stage

The fraction of inmates at secondary stage that are treated —based mainly on symptoms—, becoming susceptible, is denoted by $\phi(\mathcal{E}) \in [0,1]$ (see transition 8 in Figure A1.1). Thus, the fraction $(1-\phi(\mathcal{E}))$ of treated inmates at secondary stage acquire immunity (see transition 9 in Figure A1.1). The parameter $\phi(\mathcal{E})$ is a function of the strategy $\mathcal{E} \in \{0,1,2,3\}$, because it depends on the number of inmates at secondary stage (Y_2) that have previously been in latency stage.

In order to compute $\phi(\mathcal{E})$ we have considered the original model published in [4]. For that model, we compute the steady states at secondary, latency, remission and relapse stages. Notice that in our model remission and relapse stages are

not considered, so we are associating the remission stage with the latency, and the relapse stage to the secondary stage. Thus, in the model proposed in [4] we compute the number of individuals (at equilibrium) in the relapse stage divided by the sum of individuals in secondary and relapse stages. This fraction is considered as the proportion of individuals at secondary stage (in our model) that have previously been in the latency stage.

This procedure gives the following expression for $\phi(\mathcal{E})$:

$$\phi(\mathcal{E}) = \frac{(\sigma_{Y_2} + \tau_{Y_2}(\mathcal{E}) + \mu)(\tilde{\rho} + \tau_L(\mathcal{E}) + \mu)}{\tilde{\rho} p \sigma_{Y_2} + (\sigma_{Y_2} + \tau_{Y_2}(\mathcal{E}) + \mu)(\tilde{\rho} + \tau_L(\mathcal{E}) + \mu)}, \tag{A1.5}$$

where σ_{Y_2} and μ are described in Table A2.2 (see Appendix S2) and $\tau_{Y_2}(\mathcal{E})$ and $\tau_L(\mathcal{E})$ are given by Table A1.2. The value of $p \in [0, 1]$ corresponds to the proportion of individuals at latent stage who move toward remission stage which, according to [4] is $p = 0.25$ (see Table A2.1 in Appendix S2). Finally, $\tilde{\rho}$ is the inverse of the mean duration of the remission stage. This time, according to [4], is 6 months, so the value considered is $\tilde{\rho} = 2$ (year)$^{-1}$ (see Table A2.1 in Appendix S2).

To obtain (A1.5) we have assumed that there are no individuals entering at remission or relapse stages from outside the prison.

1.3 Flows of new inmates entering the prison

According to the model description, the rate of new inmates entering the prison is μN. This quantity has to be distributed in different stages of the disease. Given a strategy $\mathcal{E} \in \{0, 1, 2, 3\}$, the key parameter is the coverage $\xi_{\mathcal{E}} \in [0, 1]$ of the Rapid Test applied to the inmates at the entry point. Thus, if the coverage $\xi_{\mathcal{E}}$ is equal to zero (as in strategies $\mathcal{E} \in \{0, 1, 3\}$), the Rapid Test is not applied. For the strategy $\mathcal{E} = 1$, the coverage assumed is $\xi_{\mathcal{E}} = 0.8$.

The procedure at the entrance of the prison consists in application of the Rapid Test (d_1 = RT) to a proportion $\xi_{\mathcal{E}}$ of new inmates followed by a second (confirmation) test to all positive outcomes from the Rapid Test (both true and false). This confirmation test is of a non treponemal type (d_2 = VDRL). Thus, only resulting confirmed positive outcomes (i.e., after the second test) are treated. New inmates presenting the disease in any of its stages and who were not detected at the entry point, will enter to the corresponding stage in prison.

We denote the inflow rate of individuals at stage j of the disease by $\Lambda_j(\xi_{\mathcal{E}})$ (see system (A1.1) or transitions 18-23 in Figure A1.1) according to the Rapid Test coverage $\xi_{\mathcal{E}}$. More explicitely, we use the following notations: $\Lambda_S(\xi_{\mathcal{E}})$, $\Lambda_{Y_1}(\xi_{\mathcal{E}})$, $\Lambda_{Y_2}(\xi_{\mathcal{E}})$, $\Lambda_L(\xi_{\mathcal{E}})$, $\Lambda_T(\xi_{\mathcal{E}})$, and $\Lambda_I(\xi_{\mathcal{E}})$. The expressions of these values are given

by:

$$\text{(susceptibles)}\quad \Lambda_S(\xi_\mathcal{E}) = \overbrace{(1-P)\mu N}^{\text{susceptibles entering the prison}}$$
$$+ \overbrace{\xi_\mathcal{E} P\mu N \left(\alpha_{Y_1} s_{Y_1}(d_1) s_{Y_1}(d_2) + \phi(\mathcal{E})\alpha_{Y_2} s_{Y_2}(d_1) s_{Y_2}(d_2)\right)}^{\text{true positives treated}}$$

$$\text{(inmunes)}\quad \Lambda_I(\xi_\mathcal{E}) = \underbrace{(1-\xi_\mathcal{E} s_I(d_1) s_I(d_2))\alpha_I P\mu N}_{\text{immunes entering the prison}}$$
$$\left.\begin{aligned} &+ \xi_\mathcal{E} P\mu N (1-\phi(\mathcal{E}))\alpha_{Y_2} s_{Y_2}(d_1) s_{Y_2}(d_2) \\ &+ \xi_\mathcal{E} P\mu N \sum_{j\in\{L,T\}} \alpha_j s_j(d_1) s_j(d_2) \end{aligned}\right\} \text{true positives treated}$$

$$\text{(other stages)}\quad \Lambda_j(\xi_\mathcal{E}) = \underbrace{(1-\xi_\mathcal{E} s_j(d_1) s_j(d_2))\alpha_j P\mu N}_{\text{infected individuals not detected}} \qquad j \in \{Y_1, Y_2, L, T\},$$

where d_1 = RT and d_2 = VDRL.

Observe that if the Rapid Test coverage is total ($\xi_\mathcal{E} = 1$) and the applied tests perform perfectly ($s_j = 1$), all the new inmates enter to the susceptible or immune stages.

1.4 Disease dynamics under Strategies 2a, 2b, 2c, and 2d ($\mathcal{E} = 2$)

The disease dynamics when Strategy 2a, 2b, 2c, or 2d (i.e., $\mathcal{E} = 2$) is applied is given mainly by system (A1.1) (see also Figure A1.1) considering that at some instants of time $t_i \in \{t_1, t_2, \ldots, t_f\} \subset [t_0, T]$ a mass screening using the Rapid Test is applied to the existing prison population with a coverage $\tilde{\xi}_2 \in [0, 1]$. Next, a non treponemal test d_2 = VDRL is applied to confirm or discard positive outcomes (true and false) from the Rapid Test. Only those positive results from the second test are then subjected to treatment.

The value used for the coverage of the mass screening is $\tilde{\xi}_2 = 0.8$. On the other hand, the time instants where the mass screening is applied are considered to be periodic, that is $t_i = t_0 + i\Delta t$ for $i = 1, 2, \ldots, f$, where Δt is the period between two mass screening with the Rapid Test. In this study, we consider Δt equal to 1, 2, 5, and 10 years.

A mass screening at time $t_i \in \{t_1, t_2, \ldots, t_f\} \subset [t_0, T]$, with the respective confirmation test and the corresponding treatment to positive individuals, will produce an instantaneous variation in the state variables. Let us then consider the following notation: If X_j denotes one of the six state variables, then the value of this variable at time t_i is $X_j(t_i)$; moreover, we denote $X_j(t_i^+)$ the value of X_j just after the screening, confirmation test, and the corresponding treatments. Values $X_j(t_i)$ are obtained from system (A1.1). In order to determine the values $X_j(t_i^+)$ we follow an approach similar to the one described in Section 1.3, obtaining:

$$\text{(susceptibles)}\quad S(t_i^+) = \overbrace{S(t_i)}^{\text{susceptibles before mass screening}} + \overbrace{\tilde{\xi}_2\left(s_{Y_1}(d_1)s_{Y_1}(d_2)Y_1(t_i) + \phi(\mathcal{E})s_{Y_2}(d_1)s_{Y_2}(d_2)Y_2(t_i)\right)}^{\text{true positives treated}}$$

$$\text{(immunes)}\quad I(t_i^+) = \underbrace{I(t_i)}_{\text{immunes before mass screening}} + \left.\begin{aligned} &\tilde{\xi}_2(1-\phi(\mathcal{E}))s_{Y_2}(d_1)s_{Y_2}(d_2)Y_2(t_i) \\ &+ \tilde{\xi}_2 \sum_{j\in\{L,T\}} s_j(d_1)s_j(d_2)X_j(t_i) \end{aligned}\right\} \text{true positives treated}$$

$$\text{(other stages)}\quad X_j(t_i^+) = \underbrace{(1-\tilde{\xi}_2 s_j(d_1)s_j(d_2))X_j(t_i)}_{\text{infected inmates not detected}} \qquad j \in \{Y_1, Y_2, L, T\},$$

where $d_1 = \text{RT}$ and $d_2 = \text{VDRL}$.

Thus, after the procedure is finished at time t_i, the evolution of the disease follows the dynamics given by (A1.1) —but considering the new initial conditions $X_j(t_i^+)$ described above— until the next instant t_{i+1}, when the mass screening (and the corresponding procedure) is applied again.

2 Costs of interventions

In this section we present the models of costs associated with each type of intervention involved in the different strategies described in the Section *Materials and methods*. In all the interventions considered, infected individuals are detected and treated in one of the following stages of the disease: primary (Y_1), secondary (Y_2), latency (L) or tertiary (T).

We distinguish two types of interventions and their respective costs: detection and treatment inside the prison (mainly for inmates presenting symptoms), detection and treatment for individuals about to enter the prison (only valid for Strategy 1, i.e., $\mathcal{E} = 1$). However, since Strategies 2a, 2b, 2c, and 2d (i.e., $\mathcal{E} = 2$) involve jumps (or discontinuities) in the evolution of state variables due to mass interventions, we should consider the costs of detection and treatment for inmates inside the prison, as consequence of such mass screenings, in some specific instants of time. Hence, the expressions for the costs of detection and treatment inside the prison for inmates presenting symptoms will be different for Strategies 2a, 2b, 2c, and 2d.

The procedure of detection and treatment at the entrance of the prison (only strategy $\mathcal{E} = 1$), inside the prison (mainly for inmates presenting symptoms), and inside the prison due to mass screenings (only for strategy $\mathcal{E} = 2$) follows the same structure:

- A test d_1 is applied to a group of individuals;
- A confirmation test d_2 is applied to individuals resulting positive (true and false) from the first test;
- Individuals resulting positive (true and false) from the confirmation test are subjected to treatment.

For the detection inside the prison (mainly for inmates presenting symptoms), tests d_1 and d_2 are determined by the chosen strategy $\mathcal{E} \in \{0, 1, 2, 3\}$. The tests for detection at the prison entry (strategy $\mathcal{E} = 1$) or for mass screening inside the prison (strategy $\mathcal{E} = 2$) are $d_1 = \text{RT}$ and $d_2 = \text{VDRL}$.

The description of costs is organized in three sections: Costs of detection and treatment inside the prison; costs of detection and treatment at the prison entry (strategy $\mathcal{E} = 1$); and costs of strategy $\mathcal{E} = 2$. In some parts we write $X_j^{\mathcal{E}}$, for $j \in \{Y_1, Y_2, L, T\}$ to denote the respective state variables when strategy $\mathcal{E} \in \{0, 1, 2, 3\}$ is applied.

All the costs are computed for a given interval of time $[t_0, T]$, where t_0 corresponds to present time. In order to bring costs at a future time $t \geq t_0$ to present time we need to multiply by $1/(1 + r)^{(t-t_0)}$, where $r > 0$ is a given discount rate. For this purpose, if we denote

$$\varepsilon := \frac{1}{1 + r},$$

then the discount factor is $\varepsilon^{(t-t_0)}$.

The unitary costs of every test and treatment (depending on the stage of the disease) are indicated in Table A2.2 (Appendix S2). The discount rate considered

is $r = 0.03$ according to the Chilean guidelines for cost effectiveness analysis as presented in Table A2.1 in Appendix S2.

2.1 Costs of detection and treatment inside the prison

Costs of detection and treatment inside the prison (mainly for inmates presenting symptoms) depend on the strategy $\mathcal{E} \in \{0,1,2,3\}$ considered. For each strategy, there is a first test $d_1 \in \{\mathrm{VDRL}, \mathrm{RT}\}$ followed by a confirmation test $d_2 \in \{\mathrm{VDRL}, \text{FTA-Abs}\}$. This procedure is applied constantly to a group of individuals. The expression for detection costs is given by

$$C_{D_{\mathrm{inside}}}(\mathcal{E}) := C_D(d_1)\frac{(\varepsilon^{(T-t_0)}-1)}{\ln\varepsilon} \overbrace{\eta N}^{\text{group to whom } d_1 \text{ is applied}}$$
$$+ C_D(d_2)\int_{t_0}^{T} \varepsilon^{(t-t_0)} \left(\sum_{j\in\{Y_1,Y_2,L,T\}} \underbrace{s_j(d_1)\gamma_j X_j^{\mathcal{E}}(t)}_{\text{true positive of } d_1} + \underbrace{(1-e_j(d_1))(\eta N - \gamma_j X_j^{\mathcal{E}}(t))}_{\text{false positives of } d_1} \right) dt. \tag{A1.6}$$

On the other hand, the cost of treatment of individuals with positive results from the confirmation test d_2 (true and false) is given by

$$C_{T_{\mathrm{inside}}}(\mathcal{E}) := \sum_{j\in\{Y_1,Y_2,L,T\}} C_{T_j} \left(\int_{t_0}^{T} \varepsilon^{(t-t_0)} \overbrace{\tau_j(\mathcal{E}) X_j^{\mathcal{E}}(t)}^{\text{true positives}} dt \right.$$
$$\left. + \int_{t_0}^{T} \varepsilon^{(t-t_0)} \underbrace{(1-e_j(d_1))(1-e_j(d_2))(\eta N - \gamma_j X_j^{\mathcal{E}}(t))\, dt}_{\text{false positives}} \right). \tag{A1.7}$$

With expressions (A1.6) and (A1.7), the costs of strategies $\mathcal{E} \in \{0,3\}$ are given by

$$C(\mathcal{E}) := C_{D_{\mathrm{inside}}}(\mathcal{E}) + C_{T_{\mathrm{inside}}}(\mathcal{E}),$$

since strategies 0 and 3 only consider intervention inside the prison to symptomatic inmates.

2.2 Costs of detection and treatment at the prison entry

The costs of detection and treatment at the entry point are valid only for strategy $\mathcal{E} = 1$. Nevertheless, we establish these costs for a general coverage $\xi_{\mathcal{E}} \in [0,1]$ of the Rapid Test at the prison entry, recalling that $\xi_{\mathcal{E}} = 0$ when $\mathcal{E} \in \{0,2,3\}$.

The first test applied to a fraction $\xi_{\mathcal{E}} \in [0,1]$ of newly admitted inmates is $d_1 = \mathrm{RT}$. Then, a confirmation test $d_2 = \mathrm{VDRL}$ is applied to individuals giving (true and false) positive results. Therefore, the detection cost of this procedure is given by

$$C_{D_{\text{entrance}}}(\mathcal{E}) := C_D(d_1)\frac{(\varepsilon^{(T-t_0)}-1)}{\ln \varepsilon} \overbrace{\xi_{\mathcal{E}}\mu N}^{\text{group to whom } d_1 = \text{RT is applied}}$$
$$+C_D(d_2)\frac{(\varepsilon^{(T-t_0)}-1)}{\ln \varepsilon} \sum_{j\in\{Y_1,Y_2,L,T\}} \left(\underbrace{s_j(d_1)\xi_{\mathcal{E}} P\alpha_j \mu N}_{\text{true positives of RT}} + \underbrace{(1-e_j(d_1))(1-P\alpha_j)\xi_{\mathcal{E}}\mu N}_{\text{false positives of RT}} \right) \tag{A1.8}$$

where $d_1 = \mathrm{RT}$ and $d_2 = \mathrm{VDRL}$.

The cost of treatment of individuals resulting positive in the confirmation test is given by

$$C_{T_{\text{entrance}}} := \frac{(\varepsilon^{(T-t_0)}-1)}{\ln \varepsilon} \sum_{j\in\{Y_1,Y_2,L,T\}} C_{T_j} \left(\overbrace{\xi_{\mathcal{E}}\mu N P\alpha_j s_j(d_1) s_j(d_2)}^{\text{true positives}} \right.$$
$$\left. + \underbrace{(1-e_j(d_1))(1-e_j(d_2))(1-P\alpha_j)\xi_{\mathcal{E}}\mu N}_{\text{false positives}} \right). \tag{A1.9}$$

Notice that if $\xi_{\mathcal{E}} = 0$ (as in strategies $\mathcal{E} \in \{0,2,3\}$) the above costs are equal to zero.

With expressions (A1.6), (A1.7), (A1.8) and (A1.9), we can write the cost of Strategy 1 ($\mathcal{E} = 1$) as follows

$$C(\mathcal{E}) := C_{D_{\text{inside}}}(\mathcal{E}) + C_{T_{\text{inside}}}(\mathcal{E}) + C_{D_{\text{entrance}}}(\mathcal{E}) + C_{T_{\text{entrance}}}(\mathcal{E}).$$

Observe that $C_{D_{\text{entrance}}}(\mathcal{E})$ and $C_{T_{\text{entrance}}}(\mathcal{E})$ are linear with respect to the coverage $\xi_{\mathcal{E}}$ of the Rapid Test at entry. Other costs $C_{D_{\text{inside}}}(\mathcal{E})$ and $C_{T_{\text{inside}}}(\mathcal{E})$ are non linear because they depend on the state variables $X_j^{\mathcal{E}}(t)$.

2.3 Costs of Strategies 2a, 2b, 2c, and 2d

The costs of detection and treatment of Strategies 2a, 2b, 2c, and 2d (i.e., $\mathcal{E} = 2$) have the same structure of those presented in Sections 2.1 and 2.2 taking into

account that the state variables have to be updated after mass interventions at times $t_i \in \{t_1, t_2, \dots, t_f\} \subset [t_0, T]$.

Costs of detection of inmates inside the prison —procedure which is applied constantly and based mainly on symptoms—, is given by

$$\begin{aligned} C_{D_{\text{inside}}}(2) &:= C_D(d_1)\frac{(\varepsilon^{(T-t_0)}-1)}{\ln\varepsilon} \overbrace{\eta N}^{\text{group to whom } d_1 \text{ is applied}} \\ &+ C_D(d_2) \sum_{j\in\{Y_1,Y_2,L,T\}} \sum_{i=0}^{f+1} \int_{t_i}^{t_i+1} \varepsilon^{(t-t_0)} \Bigg(\underbrace{s_j(d_1)\gamma_j X_j^{\mathcal{E}}(t)}_{\text{true positives of } d_1} \\ &+ \underbrace{(1-e_j(d_1))(\eta N - \gamma_j X_j^{\mathcal{E}}(t))}_{\text{false positives of } d_1} \Bigg) dt. \end{aligned} \tag{A1.10}$$

The cost associated with treatments of inmates due to the detection procedure indicated above, is given by

$$\begin{aligned} C_{T_{\text{inside}}}(2) &:= \sum_{i=0}^{f+1} \sum_{j\in\{Y_1,Y_2,L,T\}} C_{T_j} \Bigg(\int_{t_i}^{t_{i+1}} \varepsilon^{(t-t_0)} \overbrace{\tau_j(\mathcal{E}) X_j^{\mathcal{E}}(t)}^{\text{true positives}} dt \\ &+ \int_{t_i}^{t_{i+1}} \varepsilon^{(t-t_0)} \underbrace{(1-e_j(d_1))(1-e_j(d_2))(\eta N - \gamma_j X_j^{\mathcal{E}}(t))}_{\text{false positives}} dt \Bigg). \end{aligned} \tag{A1.11}$$

In the above expressions, one has $t_{f+1} = T$. Notice also that inside the integrals in (A1.10) and (A1.11), initial conditions of state variables have to be updated to $X_j^{\mathcal{E}}(t_i^+)$, according to the dynamics of Strategies 2a, 2b, 2c, and 2d (i.e., $\mathcal{E} = 2$) described in Section 1.4, in order to integrate in intervals $[t_i, t_{i+1}]$.

The cost of mass interventions at times $t_i \in \{t_1, t_2, \dots, t_f\} \subset [t_0, T]$, for the detection part is given by

$$\begin{aligned} C_{D_{\text{mass}}}(2) &:= C_D(d_1) \overbrace{\tilde{\xi}_2 N}^{\text{group to whom } d_1 = \text{RT is applied}} \sum_{i=1}^{f} \varepsilon^{(t_i-t_0)} \\ &+ C_D(d_2) \sum_{i=1}^{f} \varepsilon^{(t_i-t_0)} \sum_{j\in\{Y_1,Y_2,L,T\}} \Bigg(\underbrace{s_j(d_1)\tilde{\xi}_2 X_j(t_i)}_{\text{true positives of RT}} + \underbrace{(1-e(d_1))(N - X_j(t_i))\tilde{\xi}_2}_{\text{false positives of RT}} \Bigg). \end{aligned} \tag{A1.12}$$

On the other hand, for the treatment part, i.e., to those positive results in the confirmation test d_2, we have

$$C_{T_{\text{mass}}}(2) := \sum_{i=1}^{f} \varepsilon^{(t_i-t_0)} \sum_{j\in\{Y_1,Y_2,L,T\}} C_{T_j} \Bigg(\overbrace{\tilde{\xi}_2 X_j(t_i) s_j(d_1) s_j(d_2)}^{\text{true positives}} \tag{A1.13}$$
$$+ \underbrace{(1-e_j(d_1))(1-e_j(d_2))(N-X_j(t_i))\tilde{\xi}_2}_{\text{false positives}} \Bigg),$$

where $d_1 = \text{RT}$ and $d_2 = \text{VDRL}$.

Thus, the cost associated with dynamics of Strategies 2a, 2b, 2c, and 2d (i.e., $\mathcal{E} = 2$) is given by

$$C(2) := C_{D_{\text{inside}}}(2) + C_{T_{\text{inside}}}(2) + C_{D_{\text{mass}}}(2) + C_{T_{\text{mass}}}(2),$$

where expressions of costs in the right-hand side are given by (A1.10), (A1.11), (A1.12) and (A1.13), respectively.

3 Health outcomes of strategies

The health outcomes associated with uninfected and infected inmates, for a given strategy $\mathcal{E} \in \{0, 1, 2, 3\}$, were assessed using QALY. For this purpose, the quality of life coefficients indicated in Table A2.2 (Appendix S2), were considered. We denote by Q_j the quality of life coefficients associated with inmates in the stage j of the disease, with $j \in \{S, Y_1, Y_2, L, T, I\}$. Thus, the health outcomes of strategies $\mathcal{E} \in \{0, 1, 3\}$ are given by

$$B(\mathcal{E}) := \int_{t_0}^{T} \varepsilon^{(t-t_0)} \Big(Q_S S^{\mathcal{E}}(t) + Q_{Y_1} Y_1^{\mathcal{E}}(t) + Q_{Y_2} Y_2^{\mathcal{E}}(t)$$
$$+ Q_L L^{\mathcal{E}}(t) + Q_T T^{\mathcal{E}}(t) + Q_I I^{\mathcal{E}}(t) \Big)\, dt,$$

where $X^{\mathcal{E}}(t)$ indicates the corresponding value of the state variable X at time t under the strategy $\mathcal{E}$.

For Strategies 2a, 2b, 2c, and 2d (i.e., $\mathcal{E} = 2$) the health outcome expression is different due to the actualization procedure of states variables described in Section

1.4. So, for $\mathcal{E} = 2$, the health outcome expression is given by

$$B(2) := \sum_{i=0}^{f+1} \int_{t_i}^{t_{i+1}} \varepsilon^{(t-t_0)} \Big(Q_S S^{\mathcal{E}}(t) + Q_{Y_1} Y_1^{\mathcal{E}}(t) + Q_{Y_2} Y_2^{\mathcal{E}}(t) + Q_L L^{\mathcal{E}}(t) + Q_T T^{\mathcal{E}}(t) + Q_I I^{\mathcal{E}}(t) \Big)\, dt,$$

where $t_{f+1} = T$. In the above expression (specifically inside the integrals) initial conditions of state variables have to be updated to $X_j^{\mathcal{E}}(t_i^+)$, according to the dynamics of Strategies 2a, 2b, 2c, and 2d (i.e., $\mathcal{E} = 2$) described in Section 1.4, in order to integrate in intervals $[t_i, t_{i+1}]$.

4 Calibration of key parameters

Most of the parameters, mainly those related to the disease dynamic and test performance, were obtained from systematic searches of indexed (PubMed, Cochrane, Scielo) and grey literature, complemented with epidemiological textbooks. We proceed with a sensitivity analysis for almost all of these parameters (see Table A2.2 in Appendix S2) according to the probability distribution indicated in the literature.

On the other hand, taking the reference values of parameters in Table A2.2 (Appendix S2) and based on a previous work that measured the syphilis prevalence in two Chilean prisons using rapid tests, we calibrate other (key) parameters in order to obtain a stationary 3% prevalence as an outcome for the Strategy 0 (current situation) (see initial conditions or the distribution by stage at the beginning of simulations in Table A2.1 in Appendix S2). We implemented this procedure in absence of historical data which has allowed an accurate model fit. The calibrated parameters were c_1 and c_2 (number of sexual partners per year for inmates in primary and secondary stages); β_1 and β_2 (probabilities of transmission of inmates in primary and secondary stages); and μ (inmates turnover, that is, μ^{-1} is the average imprisonment time). Parameters c_j and μ have an important influence in the model outcomes and they have a great variability among inmates. For this reason we decided to calibrate them. We also included parameters β_j in this calibration, because they appear multiplying c_j in the model (A1.1). While our deterministic modeling approach may not deal with the entire variability of the number of sexual partners and imprisonment times, our model is still able to present qualitatively realistic and insightful results. Indeed, the main goal of our work has been to implement a decision model for evaluating the cost-effectiveness of different interventions rather than to represent the evolution of the disease with full accuracy. In spite of the lack of available data needed to implement a more

realistic version, the simplicity of the proposed model successfully represents the desired amount of complexity observed in the disease dynamics.

Introducing the notation $\theta := (\mu, c_1, c_2, \beta_1, \beta_2)$ as the vector of parameters to be calibrated and $X^*(\theta) = (S^*, Y_1^*, Y_2^*, L^*, T^*, I^*)$ as the steady state associated with θ, we search $\theta \in [0.133, 1] \times [0, 10] \times [0, 10] \times [0, 1] \times [0, 1]$ minimizing $\|X^*(\theta) - X(0)\|^2$ (least square procedure) where $\|\cdot\|^2$ is the standard Euclidean norm in $\mathbb{R}^6$ and $X(0) = (S(0), Y_1(0), Y_2(0), L(0), T(0), I(0))$ is the vector of initial conditions indicated in Table A2.1 (Appendix S2) and obtained from (22) (the prevalence of 3%) and (7) (the distribution in different stages of the disease inside a prison). The optimal values $\theta^* = (\mu^*, c_1^*, c_2^*, \beta_1^*, \beta_2^*)$ are reported in Table A2.1 (Appendix S2).

Appendix S2. Tables of parameters: Exploratory exercise in a Chilean male prison

Table A2.1. Parameters (Part 1)

Parameter				Baseline scenario	References
Name		Nomenclature	Unit		
**Disease dynamics**	Transmission probability Y_1	β_1	-	0.627	Based on [1, 31, 29, 30, 9, 26, 4, 8][i]
	Transmission probability Y_2	β_2	-	0.605	Based on [1, 8, 31, 29, 30, 9, 26, 4][i]
	Relapse of latents in remission (proportion)	p	-	0.25	Based on [4]
	Inverse of the mean duration of remission	$\tilde{\rho}$	$(\text{years})^{-1}$	2	Based on [4]
	Proportion of individuals in Stage 1 who can transmit the infection	α	-	0.622	Based on [4][ii]
**Prison population**	Number of inmates at the beginning of simulations	N	N°	5,000	Based on [6][iii]
	Inmates turnover (1/years)	μ	N°/years	0.48	Based on [28, 24][i]
	Number of sexual partners Y_1	c_1	N°	4.098	Based on [25][i]
	Number of sexual partners Y_2	c_2	N°	4.098	
**Prevalence distribution at entry population**	Proportion of infected new inmates in Y_1	α_{Y_1}	-	0.188	Based on [21][iv]
	Proportion of infected new inmates in Y_2	α_{Y_2}	-	0.187	
	Proportion of infected new inmates in L	α_L	-	0.609	
	Proportion of infected new inmates in T	α_T	-	0.016	
	Proportion of infected new inmates in I	α_I	-	0	
**Distribution by stage at the beginning of simulations**	Number of susceptible inmates at $t = 0$	$S(0)$	N°	4,850	Based on [5][v]
	Number of inmates in Y_1 at $t = 0$	$Y_1(0)$	N°	53	
	Number of inmates in Y_2 at $t = 0$	$Y_2(0)$	N°	30	
	Number of inmates in L at $t = 0$	$L(0)$	N°	66	
	Number of inmates in T at $t = 0$	$T(0)$	N°	1	
	Number of inmates in I at $t = 0$	$I(0)$	N°	0	
**Discount rate**	Discount rate for cost effectiveness analysis	r	%	3%	Based on [22]

[i] Parameters obtained from a calibration process described in Section 4 in Appendix S1.

[ii] Stage 1 includes incubation (a separate stage in Garnett et al. [4]) and there is no transmission at this part of the stage.

[iii] Estimation based on the population incarcerated in the Santiago Sur Penitentiary Detention Center in December 2016 [6].

[iv] Based on the average distributions observed in the reported cases of syphilis in Chilean general population during the period 2007-2012 [21].

[v] Distribution of the 3% prevalence [25] based on results found in Spain by Garriga et al. For the purposes of modeling, the existence of a case in tertiary syphilis was assumed (in the study by Garriga et al. there were no cases in this stage) [5].

Table A2.2. Parameters (Part 2)

Parameter				Baseline scenario	Probabilistic Sensitivity Analysis			Referen-ces
**Name**		**Nomen-clature**	**Unit**		**Range of values**	**Distribution**	**Distribution values**	
**Duration (by stage)**	Mean duration by stage[i]	σ_{Y_1}	$(\text{years})^{-1}$	4.932	n.a.	Triangular	4.15 ; 11.41	Based on [4]
		σ_{Y_2}		3.380	n.a.	Triangular	1.87 ; 8.11	
		σ_L		0.033	n.a.	Triangular	0.03 ; 0.2	
		σ_I		0.200	n.a.	Triangular	0.2 ; 0.2	
**Prevalence at admission**	Prevalence of entrance population	P	N°/100 male population	0.76	+/-5%	Triangular	0.722; 0.798	Based on [10, 11][ii]
**Detection inside the prison over total infected inmates**	Proportion of detected inmates inside the prison over total infected inmates at each stage, with $\mathcal{E} = 0$ $\tilde{p}_j(\mathcal{E} = 0)$	$\tilde{p}_{Y_1}(\mathcal{E} = 0)$	-	0.048	n.a.	Uniform	0.048; 0.067	Based on application of surveys in Puente Alto and Arica's prisons, and [21][iii]
		$\tilde{p}_{Y_2}(\mathcal{E} = 0)$		0.083	n.a.	Uniform	0.083; 0.115	
		$\tilde{p}_L(\mathcal{E} = 0)$		0.124	n.a.	Uniform	0.124; 0.172	
		$\tilde{p}_T(\mathcal{E} = 0)$		0.276	n.a.	Uniform	0.276; 0.382	
**Prevalence inside prison**	Prevalence inside prison, by stage, with $\mathcal{E} = 0$, per 100 inmates $P_j(\mathcal{E} = 0)$	$P_{Y_1}(\mathcal{E} = 0)$	N°/100 inmates	1.05	+/-5%	Triangular	1.001 ; 1.106	Based on [25, 5] and exams applied in Puente Alto prison[iv]
		$P_{Y_2}(\mathcal{E} = 0)$		0.61	+/-5%	Triangular	0.576 ; 0.637	
		$P_L(\mathcal{E} = 0)$		1.32	+/-5%	Triangular	1.258 ; 1.391	
		$P_T(\mathcal{E} = 0)$		0.02	+/-5%	Triangular	0.015 ; 0.017	
**Sensitivity** $s_X(d)$	Rapid Test - RT (SD Bioline Syphilis 3.0)	$s_{Y_1}(\text{RT})$	%	84.33%	n.a.	Normal	$\mu = 84.33\%$; $\sigma = 0.95\%$	Based on [7, 12, 14, 13, 15, 32, 17, 33]
		$s_{Y_2}(\text{RT})$		84.33%	n.a.	Normal	$\mu = 84.33\%$; $\sigma = 0.95\%$	
		$s_L(\text{RT})$		84.33%	n.a.	Normal	$\mu = 84.33\%$; $\sigma = 0.95\%$	
		$s_T(\text{RT})$		84.33%	n.a.	Normal	$\mu = 84.33\%$; $\sigma = 0.95\%$	
	VDRL	$s_{Y_1}(\text{VDRL})$	%	70.74%	n.a.	Normal	$\mu = 70.74\%$; $\sigma = 3.32\%$	Based on [34, 16, 2, 20, 27]
		$s_{Y_2}(\text{VDRL})$		96.69%	n.a.	Normal	$\mu = 96.69\%$; $\sigma = 1.63\%$	
		$s_L(\text{VDRL})$		92.86%	n.a.	Normal	$\mu = 92.86\%$; $\sigma = 3.97\%$	
		$s_T(\text{VDRL})$		69.23%	n.a.	Normal	$\mu = 69.23\%$; $\sigma = 7.39\%$	
	FTA-ABS	$s_{Y_1}(\text{FTA-ABS})$	%	97.40%	n.a.	Normal	$\mu = 97.40\%$; $\sigma = 1.19\%$	Based on [20, 27]
		$s_{Y_2}(\text{FTA-ABS})$		97.40%	n.a.	Normal	$\mu = 97.40\%$; $\sigma = 1.19\%$	
		$s_L(\text{FTA-ABS})$		97.40%	n.a.	Normal	$\mu = 97.40\%$; $\sigma = 1.19\%$	
		$s_T(\text{FTA-ABS})$		97.40%	n.a.	Normal	$\mu = 97.40\%$; $\sigma = 1.19\%$	

**Specificity** $e_X(d)$	RT	e_{Y_1}(RT)	%	98.69%	n.a.	Normal	$\mu = 98.69\%$; $\sigma = 0.16\%$	Based on [7,12,14,13,15,32,17,33]
		e_{Y_2}(RT)		98.69%	n.a.	Normal	$\mu = 98.69\%$; $\sigma = 0.16\%$	
		e_L(RT)		98.69%	n.a.	Normal	$\mu = 98.69\%$; $\sigma = 0.16\%$	
		e_T(RT)		98.69%	n.a.	Normal	$\mu = 98.69\%$; $\sigma = 0.16\%$	
	VDRL	e_{Y_1}(VDRL)	%	80.72%	n.a.	Normal	$\mu = 80.72\%$; $\sigma = 1.53\%$	Based on [34,16,2,20,27]
		e_{Y_2}(VDRL)		80.72%	n.a.	Normal	$\mu = 80.72\%$; $\sigma = 1.53\%$	
		e_L(VDRL)		80.72%	n.a.	Normal	$\mu = 80.72\%$; $\sigma = 1.53\%$	
		e_T(VDRL)		80.72%	n.a.	Normal	$\mu = 80.72\%$; $\sigma = 1.53\%$	
	FTA-ABS	e_{Y_1}(FTA -ABS)	%	99.23%	n.a.	Normal	$\mu = 99.23\%$ $\sigma = 0.44\%$	Based on [20,27]
		e_{Y_2}(FTA -ABS)		99.23%	n.a.	Normal	$\mu = 99.23\%$; $\sigma = 0.44\%$	
		e_L(FTA -ABS)		99.23%	n.a.	Normal	$\mu = 99.23\%$; $\sigma = 0.44\%$	
		e_T(FTA -ABS)		99.23%	n.a.	Normal	$\mu = 99.23\%$; $\sigma = 0.44\%$	
**Cost**[v] (C)	Diagnosis and confirmation test $C_D(d)$	C_D(VRDL)[vi]	US$	$11.10	+/-10%	Gamma	a=100; b=72.06	Based on FONASA tariffs [3] and CENABAST
		C_D(FTA -ABS)[vi]		$13.32	+/-10%	Gamma	a=100; b=86.46	
		C_D(RT)[vii]		$2.98	+/-10%	Gamma	a=100; b=19.35	Based on manufacturer price[vii]
	Treatment (by stage) $C_{T,X}$	C_{T,Y_1}		$55.65	+/-10%	Gamma	a=100; b=361.32	Based on protocols [20,18,19,23], interviews to keys informants, FONASA tariffs [3] and CENABAST prices[viii]
		C_{T,Y_2}		$74.89	+/-10%	Gamma	a=100; b=486.28	
		$C_{T,L}$		$86.33	+/-10%	Gamma	a=100; b=560.58	
		$C_{T,T}$		$99.49	+/-10%	Gamma	a=100; b=646.04	
**Quality of Life Coefficient** (Q)	Coefficient by stage Q_X	Q_S	QALY	1	n.a.	Beta	a=1.711; b=0.675	Based on application of the EQ-5D questionnaire and profiles of Superintendencia de Salud's study [35]
		Q_{Y_1}		0.737	n.a.	Beta	a=1.711; b=0.675	
		Q_{Y_2}		0.737	n.a.	Beta	a=1.711; b=0.675	
		Q_L		0.737	n.a.	Beta	a=1.711; b=0.675	
		Q_T		0.737	n.a.	Beta	a=1.711; b=0.675	
		Q_I		1	n.a.	Beta	a=1.711; b=0.675	
**Diagnosis coverage $t = 0$ situation**	Proportion of inmates undergoing diagnosis inside the prison	η	-	0.0049	+/-5%	Triangular	0.0047; 0.0051	Based on administrative records of Gendarmería de Chile[ix]

[i] Primary stage duration considers merging primary and incubation stages included in Garnett et. al [4].

[ii] Weighted average prevalence of general syphilis in men from 20 to 49 years old in Chile 2015, according to estimated prevalence of the IHME [10] and estimated population [11].

[iii] Based on % of infected people who reported having a diagnosis in surveys conducted in Puente Alto and Arica's prisons. Stage distribution is based on the average distributions observed in the reported cases of syphilis in Chile during the period 2007-2012 [21].

[iv] A prevalence of 3% was assumed based on the results obtained in Arica prison [25] and exams applied in Puente Alto prison. Stage distribution is based on results found in Spain by Garriga et al. For the purposes of modeling, the existence of a case in tertiary syphilis was assumed (in the study by Garriga et al. there were no cases in this stage) [5].

[v] US$ 2017.

[vi] Includes costs for sample collection and medical consultation.

[vii] Includes costs for sample collection.

[viii] The cost of treatment according to protocol, the expected cost for re-treatment and treatment of neurosyphilis and post-treatment controls are included for all stages. For latent syphilis, treatment associated with early and late latent syphilis is weighted.

[ix] Based on the average proportion of inmates undergoing diagnosis test of syphilis, inside the Santiago Sur Penitentiary Detention Center in the period 2011-2013 (Administrative Record of Gendarmería de Chile).

References

1. L. J. Alexander and A. G. Schoch. Prevention of syphilis: Penicillin calcium in oil and white wax, u. s. p., bismuth ethylcamphorate and oxophenarsine hydrochloride in treatment, during incubation stage, of persons exposed to syphilis. *Archives of Dermatology and Syphilology*, 59(1):1–10, 01 1949.
2. P. P. Bosshard. Usefulness of igm-specific enzyme immunoassays for serodiagnosis of syphilis: Comparative evaluation of three different assays. *Journal of Infection*, 67(1):35 – 42, 2013.
3. FONASA. Arancel modalidad institucional MAI 2017 [internet]. available from: https://www.fonasa.cl/sites/fonasa/prestadores/normativa/aranceles, 2017.
4. G. Garnett, S. Aral, D. Hoyle, W. Cates, and R. Anderson. The natural history of syphilis. implications for the transmission dynamics and control of infection. *Sexually transmitted diseases*, 24:185–200, 5 1997.
5. C. Garriga, P. Gómez-Pintado, M. Diez, E. Acín, and A. Díaz. Characteristics of cases of infectious syphilis diagnosed in prisons, 2007-2008. *Revista española de sanidad penitenciaria*, 13:52–7, 10 2011.
6. Gendarmería de Chile. Estadística de población penal atendida a diciembre de 2016, 2017.
7. A. J. Herring, R. C. Ballard, V. Pope, R. A. Adegbola, J. Changalucha, D. W. Fitzgerald, E. W. Hook, A. Kubanova, S. Mananwatte, J. W. Pape, A. W. Sturm, B. West, Y. P. Yin, and R. W. Peeling. A multi-centre evaluation of nine rapid, point-of-care syphilis tests using archived sera. *Sexually Transmitted Infections*, 82(suppl 5):v7–v12, 2006.
8. D. L. Heymann. *Control of communicable diseases manual*. American Public Health Association, 20th edition, 2015.

9. E. W. Hook and C. M. Marra. Acquired syphilis in adults. *New England Journal of Medicine*, 326(16):1060–1069, 1992. PMID: 1549153.
10. Institute for Health Metrics and Evaluation. Epi visualization, 2017.
11. Instituto Nacional de Estadísticas de Chile. Proyecciones de población chile 2015, 2017.
12. Y. Jafari, R. W. Peeling, S. Shivkumar, C. Claessens, L. Joseph, and N. P. Pai. Are treponema pallidum specific rapid and point-of-care tests for syphilis accurate enough for screening in resource limited settings? evidence from a meta-analysis. *PloS one*, 8(2), 2013.
13. K. Lee, H. Park, E. Y. Roh, S. Shin, K. Park, M. Park, and E. Song. Characterization of sera with discordant results from reverse sequence screening for syphilis. *BioMed research international*, 2013:269347, 03 2013.
14. J Li, H-Y Zheng, L-N Wang, Y-X Liu, X-F Wang, and X-R Liu. Clinical evaluation of four recombinant treponema pallidum antigen-based rapid diagnostic tests for syphilis. *Journal of the European Academy of Dermatology and Venereology*, 23(6):648–650, 2009.
15. D. Mabey, R. W. Peeling, R. Ballard, A. S. Benzaken, E. Galbán, J. Changalucha, D. Everett, R. Balira, D. Fitzgerald, P. Joseph, S. Nerette, J. Li, and H. Zheng. Prospective, multi-centre clinic-based evaluation of four rapid diagnostic tests for syphilis. *Sexually Transmitted Infections*, 82(suppl 5):v13–v16, 2006.
16. K. Manavi, H. Young, and A. McMillan. The sensitivity of syphilis assays in detecting different stages of early syphilis. *International Journal of STD & AIDS*, 17(11):768–771, 2006. PMID: 17062183.
17. B. Mehra, S. Bhattar, S. Saxena, D. Rawat, and P. Bhalla. Evaluation of sd bioline syphilis 3.0 for rapid diagnosis of syphilis: Report from a regional sexually transmitted infection reference laboratory in north india. *Journal of Laboratory Physicians*, 8:36, 01 2016.
18. Ministerio de Salud de Chile. Normas de manejo y tratamiento de infecciones de transmisión sexual (ITS). norma general técnica N°103 del ministerio de salud. aprobada por decreto exento N°424 del 17 de julio de 2008., 2008.
19. Ministerio de Salud de Chile. Norma conjunta de prevención de la transmisión vertical del VIH y sífilis. norma general técnica N°0141 del 2012., 2012.
20. Ministerio de Salud de Chile. Regula el uso de técnicas de laboratorio para el apoyo al diagnóstico y seguimiento de sífilis en usuarios/as (no donantes) y establece criterios de organizació y registros. circular N°13 del 23 de septiembre de 2015, 2015.
21. Ministerio de Salud de Chile. Base de datos de notificación obligatoria (ENO) de sífilis. Chile 2007-2012., 2016.
22. Ministerio de Salud de Chile. Guía metodológica para la evaluación económica de intervenciones en salud en Chile., 2016.
23. Ministerio de Salud de Chile. Normas de profilaxis, diagnóstico y tratamiento de las infecciones de transmisión sexual (ITS). norma general técnica N°187 aprobada por resolución exenta N°484 del 20 de mayo de 2016., 2016.
24. A.M. Morales, N. Muñoz, G. Welsch, and Fábrega J. La reincidencia en el sistema penitenciario chileno. *Fundación Paz Ciudadana - Universidad Adolfo Ibáñez*, page 192, 2012.
25. M. Nájera De Ferrari, X. Aguilera Sanhueza, C. González, A.M. Olea, I. Delgado Becerra, M.B. De Gregorio Rebeco, and et al. La carga oculta de sífilis en personas privadas de libertad: medición de prevalencia con test rápido en cárcel de arica. *Medwave*, 16(Suppl 6):2–4, 2016.
26. K.E. Nelson and C.M. Williams. *Infectious Disease Epidemiology: Theory and Practice*. Infectious Disease Epidemiology: Theory and Practice. Jones & Bartlett Learning, 2014.

27. S. Ratnam. The laboratory diagnosis of syphilis. *The Canadian journal of infectious diseases & medical microbiology. Journal canadien des maladies infectieuses et de la microbiologie médicale / AMMI Canada*, 16:45–51, 02 2005.
28. M. Sánchez and D. Piñol. Condiciones de vida en los centros de privación de libertad en Chile. *Santiago de Chile: Instituto de Asuntos Públicos, Universidad de Chile*, page 49, 2015.
29. P. C. Schober, G. Gabriel, P. White, W. F. Felton, and R. N. Thin. How infectious is syphilis? *Sexually Transmitted Infections*, 59(4):217–219, 1983.
30. D. Schrijvers, R. Josse, A. Trebucq, A. Dupont, H. Cheringou, and B. Larouzé. Transmission of syphilis between sexual partners in gabon. *Sexually Transmitted Infections*, 65(2):84–85, 1989.
31. A. L. Schroeter, R. H. Turner, J. B. Lucas, and W. J. Brown. Therapy for incubating syphilis: Effectiveness of gonorrhea treatment. *JAMA*, 218(5):711–713, 11 1971.
32. P. Smit, D. Mabey, J. Changalucha, J. Mngara, B. Clark, A. Andreasen, J. Todd, M. Urassa, B. Zaba, and R. Peeling. The trade-off between accuracy and accessibility of syphilis screening assays. *PloS one*, 8:e75327, 09 2013.
33. F. Yaya Bocoum, H. Ouedraogo, G. Tarnagda, A. Kiba, S. Tiendrebeogo, F. Bationo, B. Liestman, S. Diagbouga, C. Zarowsky, R. Traoré, and S. Kouanda. Evaluation of the diagnostic performance and operational characteristics of four rapid immunochromatographic syphilis tests in burkina faso. *African health sciences*, 15:360–7, 06 2015.
34. H. Young, J. Pryde, L. Duncan, and J. Dave. The architect syphilis assay for antibodies to treponema pallidum: an automated screening assay with high sensitivity in primary syphilis. *Sexually Transmitted Infections*, 85(1):19–23, 2009.
35. V. Zarate, P. Kind, P. Valenzuela, A. Vignau, P. Olivares-Tirado, and A. Munoz. Social valuation of eq-5d health states: The chilean case. *Value in Health*, 14(8):1135 – 1141, 2011.
